# A Novel Heterozygous Mutation of the *COL4A3* Gene Causes a Peculiar Phenotype without Hematuria and Renal Function Impairment in a Chinese Family

**DOI:** 10.1155/2019/8705989

**Published:** 2019-02-10

**Authors:** Liang Xia, Yangjia Cao, Yang Guo, Guangyi Ba, Qiong Luo, Haibo Shi, Yanmei Feng, Shankai Yin

**Affiliations:** ^1^Department of Otolaryngology, Shanghai Jiao Tong University Affiliated Sixth People's Hospital, No. 600, Yishan Road, Xuhui District, 200233 Shanghai, China; ^2^Metabolic Bone Disease and Genetic Research Unit, Department of Osteoporosis and Bone Diseases, Shanghai Jiao Tong University Affiliated Sixth People's Hospital, 600 Yi-Shan Rd, Shanghai 200233, China

## Abstract

Mutations in the *COL4A3* gene are frequently reported to be associated with various types of hereditary nephropathy. *COL4A3* encodes the *α*3 chain of type IV collagen, which is the main structural protein in the basement membrane. Mutations in this gene are always related to kidney performance, and deafness and ocular lesion have also been reported. In this study, using next-generation sequencing, we investigated the DNA of a family visiting a clinic for hearing loss. A new missense mutation was found in *COL4A3* of 5 patients, c.3227C>T (p.P1076L). Based on these results, we predict that the mutation is pathogenic and leads to abnormal collagen IV. Here, we report for the first time on this autosomal dominant syndrome, characterized by hearing loss and eye abnormalities, but without renal damage, in all carriers. Since the oldest patient in the trial was less than 50 years old, however, we recommend that renal examination be reviewed regularly. Our results reveal expansion in the mutation spectrum of the *COL4A3* gene and phenotypic spectrum of collagen IV disease. Our study suggests that next-generation sequencing is an economical and effective method and may help in the accurate diagnosis and treatment of these patients.

## 1. Introduction

Type IV collagen, the major structural component of basement membranes (BMs), is a multimeric protein composed of 3 *α* subunits. These subunits are encoded by 6 different genes, *α* 1 through *α* 6, each of which can form a triple helix structure with 2 other subunits to form type IV collagen [1]. Trimers composed of *α*3, *α*4, and *α*5 chains are restricted in expression to specific BMs of the kidney, inner ear, and eye [2]. The *COL4A3* gene encodes *α*3 chains, and it is concluded that pathogenic *COL4A3* mutations account for Alport syndrome (AS) [3]. Variants in the *COL4A3* gene also cause diabetic kidney disease from maturity-onset diabetes in the young and those with familial benign hematuria (FBH) [4]. In addition, recent studies support that the *COL4A3* gene is a newly discovered pathogenic gene related to focal segmental glomerulosclerosis (FSGS) [5–7]. Autosomal dominant Alport syndrome causes hematuria, proteinuria, or progressive kidney disease. It also can be accompanied by late-onset high-tone sensorineural hearing loss and/or ocular abnormalities [8]. Hearing loss is directly related to the progression of renal failure in Alport syndrome [9]. The characteristics of FBH are persistent or recurrent hematuria without proteinuria, renal failure, or extra renal symptoms [10]. FSGS is a kind of proteinuria and end-stage renal disease (ESRD) associated with glomerular tissue pathological changes [11].

Recently, we enrolled a Chinese family on the clinic with a new mutation in the *COL4A3* gene. Profound hearing loss and retinitis pigmentosa (RP) were found in pathogenic gene carriers, but kidney impairment was not present. Mutations in the *COL4A3* gene that often lead to more atypical phenotypes have received increasing attention, because clarifying the new pathogenesis can promote the development of new treatment methods and create new areas of research on hearing impairment. Targeted next-generation sequencing (NGS) was used to identify the genetic cause of deafness in this family.

## 2. Materials and Methods

### 2.1. Subjects

The program was approved by the Ethics Committee of Shanghai Jiao Tong University Affiliated Sixth People's Hospital. The present study recruited all 7 family members. In addition, 250 healthy donors were recruited as controls for a mutation analysis. All participants involved in the study were of Han ethnicity and provided written informed consent prior to the study's commencement.

### 2.2. Phenotype Evaluation

The hearing levels of all participating members were measured by pure tone audiometry or auditory brainstem response. Ophthalmologic examination included fundus photography, a visual field test, and an electroretinogram. Through physical and radiological examination, pathological changes in the middle ear were excluded as were the presence of cochlear lesions and vestibular aqueduct syndrome. Additional auditory evaluations included otoscopic examination, an otoacoustic emissions test, and temporal bone high-resolution computed-tomography scanning. Examinations also included ophthalmologic exams, routine urine exam, urine protein quantitation, and blood biochemical analysis (retinal and liver function) of all family members.

### 2.3. Genetic Analysis

To make a precise diagnosis, we performed targeted NGS of related deafness genes. The collection of 5 mL peripheral blood samples taken from individuals was used for the following experiment. A capture panel (NimbleGen, Madison, USA) of deafness genes had been previously designed and assessed by our group. The capture panel included all exons together with the flanking exon and intron boundaries (±15 bp) of 180 genes. Genomic DNA of each subject was extracted using a QIAamp DNA Blood Midi Kit (Qiagen, Hilden, Germany). The DNA was then fragmented by sonication using a Covaris LE220 (Woburn, Massachusetts, USA) to generate a paired-end library (200–250 bp). The library was enriched by array hybridization [12]. The magnitude of enrichment of the products was then evaluated with an Agilent 2100 Bioanalyzer (Santa Clara, California, USA) and ABI StepOne™ real-time PCR system (Applied Biosystems, Foster, CA, USA). Then, captured library sequencing was carried out using a BGISEQ-500 analyzer (BGI, Shenzhen, China) following the manufacturer's standard sequencing protocols to generate paired-end reads. Image processing and base calling were executed using Illumina Pipeline software (version 1.3.4) to generate raw data.

### 2.4. Mutational Analysis

We performed bioinformatics processing and analyzed the data after receiving the raw data. Previously published filtering criteria were used to generate “clean reads” for further analysis [12]. The “clean reads” (with a length of 90 bp) originated in targeted sequencing and filtering were then aligned to the human genome reference (hg19) using the BWA (Burrows-Wheeler Aligner) Multi-Vision software package [13]. We used SOAPsnp software [14] and SAMtools [15] to detect SNVs and indels, respectively. All SNVs and indels were filtered and estimated through cross-referencing against multiple databases, including NCBI dbSNP, HapMap, 1000 Genomes project, and a database containing DNA from 250 healthy Chinese adults. We used the following three different bioinformatics algorithms to predict the variants, including missense mutations and frame shift mutations: Polyphen-2 (http://genetics.bwh.harvard.edu/pph2/), SIFT (http://sift.jcvi.org/), and Mutation Master (http://www.Mutationtaster.org/). Furthermore, amino acid conservation was analyzed using the UniProt database (https://www.uniprot.org/).

### 2.5. Sanger Sequencing

Candidate gene mutations were validated using conventional Sanger sequencing methods. The primers were designed using the online Primer3 software. The PCR products were purified by shrimp alkaline enzyme (SAP) (from Promega) and exogenous enzyme I (EXO I) (from Epicentre) and sequenced using a BigDye® Terminator Cycle Sequencing Ready Reaction Kit, version 3.1 (Foster, CA, USA). An ABI 3730xl sequencer (Carlsbad, CA, USA) was used to analyze the sequencing products. The sequencing documents were analyzed by PolyPhred software.

## 3. Results

### 3.1. Clinical Phenotype

**Figure 1:**
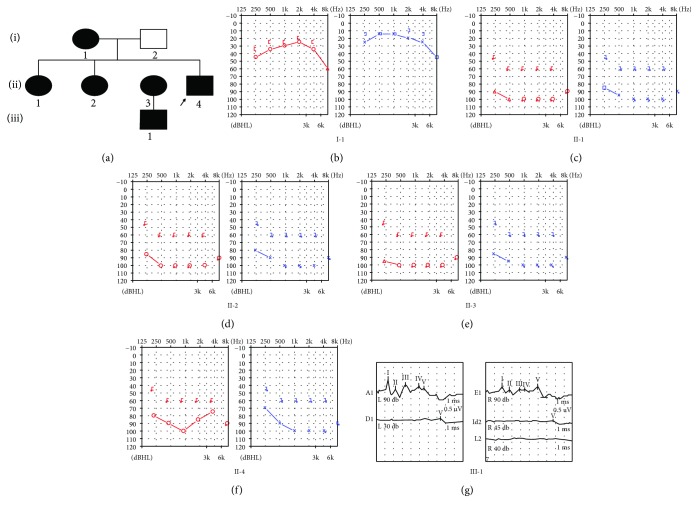
(a) Pedigree of a family with the COL4A3 mutation. Affected family members are shown in black symbols. The arrow identifies the proband in the family. (b–f) Audiogram of the affected members in the family. The red mark represents the right ear, and the blue mark represents the left ear. (g) Result of auditory brainstem response in III-1. The results show that the child's hearing is normal.

**Figure 2:**
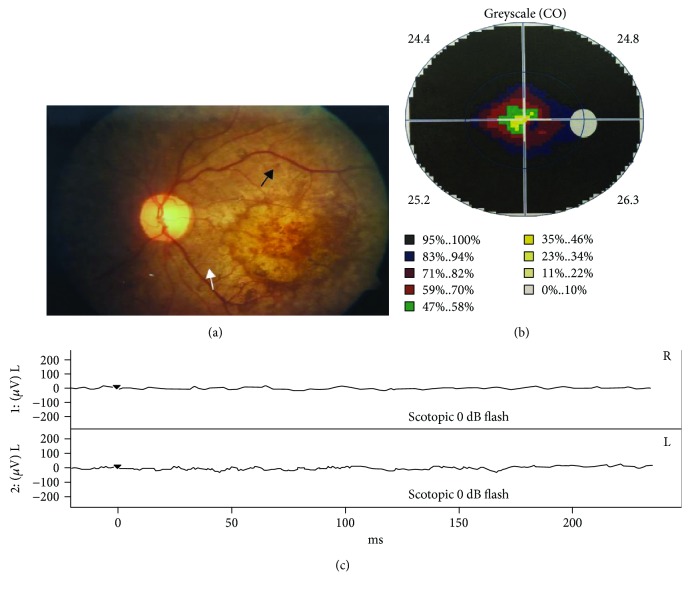
(a) Retina (right) reveals a retinopathy pigmented with attenuated retinal arterioles (white arrows). The midperiphery reveals bone spicule-like pigmentation (black arrows). (b) Reduction of the visual field. (c) An electroretinogram indicated dysfunction in the binocular retina.

Figure 1 shows the audiograms of family members. The proband and his sister had bilateral hearing loss that began in their first decade and gradually developed to severe hearing loss in the second decade. The proband's mother had mild hearing loss while his father was completely normal. No inner ear malformation of any subject was found by temporal bone high-resolution computed-tomography scanning. Ophthalmoscopy demonstrated retinal bone spicule pigmentary changes, retinal vessel attenuation, and diffuse macular atrophy in both eyes of the proband (Figure 2(a)). The proband and his sister also suffered from night vision disturbance and reduction of the visual field (Figure 2(b)). The proband and his sisters had an established clinical diagnosis of RP (Figure 2(c)). However, the proband's mother was unaffected by RP, but she was nyctalopic. But son (III-1) of II-3, a 2-year-old male, had normal phenotypes in the ear, eye, and kidney. All family members received routine blood and urine tests (including urinary sediment quantification), which showed no hematuria, proteinuria, or impaired renal function. Clinical characteristics are presented in Table 1 for all members.

### 3.2. Identification of Pathogenic Mutations in *COL4A3*

**Figure 3:**
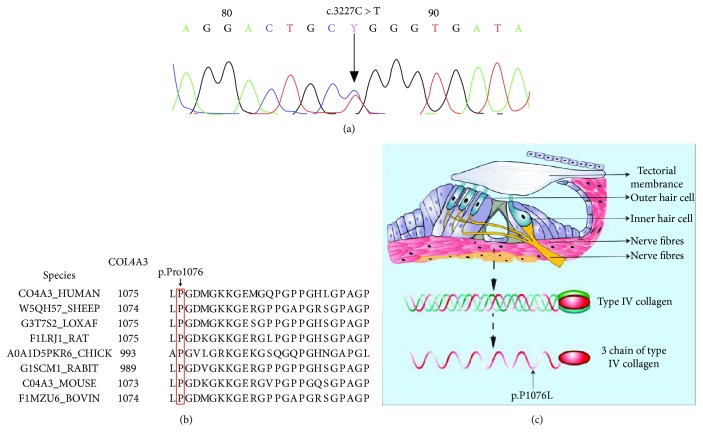
(a) Heterozygous mutation from C to T in exon 38 of COL4A3 identified by sequence analysis. (b) Conservation analysis of the collagen type IV *α*3 chain p.Pro1076 amino acid residue. The result shows that the amino acids at this site are highly conservative, and the mutant amino acids might have a great influence on structure and function. (c) Schematic diagram of the molecular structure of type IV collagen in the organ of Corti and amino acid mutation sites in the *α*3 chain of type IV collagen. Type IV collagen is abundantly expressed in the basement membrane, and mutation will affect the structure and function of the whole cochlea.

Sequence analysis of DNA in this pedigree identified a novel heterozygous mutation: C to T in exon 38 of *COL4A3* (NM_000091, c.3227C>T), which led to an amino acid substitution (p.Pro1076Leu) (Figure 3(a)). The missense mutation in *COL4A3* was absent in the 250 healthy controls. The mutation was found in I-1, II-1, II-2, II-3, II-4, and III-1, consistent with an autosomal dominant mode of inheritance.

### 3.3. Bioinformatics Analysis of the *COL4A3* Mutation

The variant sequence was highly conserved across different species (Figure 3(b)). To further evaluate the possible deleterious consequence of the novel COL4A3 missense mutation, three different in silico algorithms were used. SIFT analysis classified the variant as “intolerance” with a score of 0.01 (the threshold for intolerance is 0.05). The PolyPhen-2 software predicted the p.P1076L substitution to be probably damaging, with a score of 0.732. Additionally, the MutationTaster software supported the pathogenic effect of the variant, which was reported to be “disease-causing.” These results suggest that the novel variant would substantially alter the function of the resulting protein.

## 4. Discussion

In this study, we identified a novel mutation (c.3227C>T) in *COL4A3*. Mutations in *COL4A3* always lead to kidney-related pathogenic phenotypes, but the family in the study presented recognized diagnostic challenges in kidney disease, including atypical clinical features. Increasing evidence has shown that identical variants in *COL4A3* are benign, but related to autosomally recessive AS, FSGS, and FBH [1, 5, 10]. The transmission of AS can be autosomal dominant, autosomal recessive, or X-linked, and the prognosis is poor. On the contrary, BFH showed a good autosomal dominant pattern of prognosis [16]. All *COL4A3* mutations in previous research are summarized in Table 2. The age of onset is inconsistent. Most patients had the renal and extra renal phenotype, while others had only the renal phenotype. Rosado et al. found another *COL4A3* heterozygous mutation, which, in a few of the carriers, was also associated with a lack of kidney disease, but there was mild deafness [9]. However, a particular *COL4A3* mutation without renal damage in all carriers has not been reported. The proband and his sister in this study had severe hearing loss and eye abnormalities, but no renal impairments, such as hematuria and proteinuria, were found. In addition, the III-1, 2-year-old male, had no abnormality phenotype. We hoped that the ultrastructure of the glomerular basement membrane could be examined by renal biopsy, but this diagnostic test was refused.

Type IV collagen is usually an extracellular structural protein and constitutes a collagen branch network, which is an important component of BMs [1]. The type IV collagen molecule consists of three chains, each of which consists of three similar components: (1) a “7S” domain at the amino terminus, (2) a long collagen domain of about 1400 Gly-X-Y repeats interrupted by a short non-collagen region and forming a triple helix with the other two chains—the X and Y positions are usually proline and hydroxyproline but also can be any residue—and (3) a non-collagen “NC1” domain of about 230 residues at the carboxyl terminus, folded into a globular structure. *COL4A3* is located on 2q36-37, encoding an *α*3 collagen chain of type IV collagen [5, 27].  Figure 3 shows a schematic diagram of the molecular structure of type IV collagen in the organ of Corti and amino acid mutation sites of the *α*3 chain of type IV collagen in our study.

Even if environmental factors and different lifestyles influence the characteristics of disease in different family members [21], protein structure affected by mutation is the most important. The mechanism by which the mutation affects the function of *COL4A3* is not fully understood, yet the different clinical manifestations of the *COL4A3* mutation can be explained in this way. By integrating the altered collagen chain into the final network, normal structure and function are disrupted [28].

The *COL4A3* mutation in this family is located in the collagen domain. Proline can form an alpha helix structure in protein, destroying the *α*-helix, which will destroy the collagen skeleton. Type IV collagen *α*3, *α*4, and *α*5 chains are known to aggregate in the endoplasmic reticulum (ER) to form helical heterotrimers. Mutated *COL4A3* chains retained in the ER are associated with activation of the UPR pathway, leading to cytotoxicity and apoptosis [29]. Synthesis of the same amount of normal and abnormal chains will lead to the 1 : 1 ratio between the abnormal and normal molecules. Abnormal collagen homopolymers may cause the molecular folding, secretion, and extracellular matrix formation [30]. Other similar studies have also mentioned that misfolded proteins can be secreted into BM or accumulate in podocytes, disrupting glomerular selective barrier properties and activating downstream pathological pathways [31, 32]. Zehnder et al. discovered that in human cochlea, the *α*3 chain is specifically expressed in collagen bundles in the basement membrane, spiral ligament, and spiral margin. These results suggest that hearing loss may be due to cochlear micromechanical changes or are consistent with the helical ligament dysfunction hypothesis [2]. Type IV collagen *α*3 chains are also found in the basement membranes of the conjunctiva, cornea, iris, lens capsule, and Descemet's and Bruch's membrane [33]. Therefore, mutation in the COL4A3 gene leads to structural and functional disorders of type 4 collagen in the affected basement membrane. Ultimately, it leads to abnormal ocular phenotypes.

The special phenotype of *COL4A3* in this study may also be due to haploinsufficiency [34]. Studies have shown that the heterozygous *COL4A3* mutation can lead to a decrease in expression [4]. A mutant gene in an allele causes the amount of protein encoded by a heterozygous mutant gene to fail to reach the threshold required for the normal functioning of the protein encoded by the two alleles thus causing the protein encoded by the heterozygous mutant gene to fail to perform its normal physiological function, which is functionally equivalent to a nonsense mutation [35]. If haploinsufficiency is a mechanism by which *COL4A3* mutations affect gene expression regulation, the kidney can avoid the effect of partial loss of *COL4A3* function, because COL4A3 may be expressed at a much higher level in the kidney than in the cochlea and eye [36, 37]. Thus, COL4A3 levels may be sufficient to maintain kidney function, which may not be the case in the cochlea and eyes that exhibit much lower levels of expression. Alternatively, the mutant COL4A3 protein may lose some of its functions, but not others, rather than just lowering the overall level of COL4A3 function. If the key function is related to the cochlea and the eye, but not to the kidney, the mutation can affect the cochlea and the eye, but not the kidney.

One of the important specificities of the heterozygous *COL4A3* mutation reported here is that the renal phenotype was not found in I-1, II-1–II-4, and III-1. The wide phenotypic transformation with *COL4A3* mutations and the presence of incomplete penetrance suggest that a simple Mendelian model is inadequate to explain the genetic mechanism of the disease [38].

## 5. Conclusion

In summary, the study expanded the phenotypic spectrum of *COL4A3* mutation carriers. It is noteworthy that although we have found a heterozygous *COL4A3* mutation (c.3227 C>T) that may cause extreme deafness and ocular abnormalities, we should continue to monitor possible delayed kidney disease. Further, the autosomal dominant pattern in this case suggests to us the importance of this mode to deafness and ocular abnormalities, though 70 to 85% of the mode of inheritance of neurosensory hearing impairment occurs in an autosomal recessive pattern. Moreover, the novel pathogenic mutation identified in this study will be important for carrier testing and premarital screening for this family and their relatives in the future.

## Figures and Tables

**Table 1:** Clinical and genetic data for the family with the *COL4A3* c.3227C>T (p.P1076L) mutation.

Subject	I-1	I-2	II-1	II-2	II-3	II-4	III-1
Age	47	50	20	21	22	16	2
Gender	F	M	F	F	F	M	M
Hearing loss	Mild	Normal	Profound	Profound	Profound	Profound	Normal
Hematuria	No	No	No	No	No	No	No
Microhematuria	No	No	No	No	No	No	No
Proteinuria (g/24 h)	0.095	0.099	0.100	0.106	0.120	0.059	0.008
Creatinine (*μ*moI/L)	54	66	58	67	59	73	27
Renal failure	No	No	No	No	No	No	No
Nyctalopia	Yes	No	Yes	Yes	Yes	Yes	No
Visual field loss	No	No	Yes	Yes	Yes	Yes	No
Pigmented retinopathy	No	No	Yes	Yes	Yes	Yes	No
Genotype	Heterozygote	Normal	Heterozygote	Heterozygote	Heterozygote	Heterozygote	Heterozygote

M/F: male/female.

**Table 2:** The clinical characteristics of patients carrying *COL4A3* mutations.

References	Populations	Age	Gender	Mutation state	Mutation	Hematuria	Proteinuria	Hearing loss	Ocular lesions
[16]	UK	47	M	Heterozygous	c.3418+1G>T c.4664C>T;	+	+	+	+
[17]	Spanish	58	M	Heterozygous	c.998G>A;	+	+	+	—
[18]	Sri Lankan	14	F	Homozygous	c.1219G>T; c.1223_1224delGG	+	+	+	—
[9]	Spanish	32	F	Heterozygous	c.345 delG;	+	+	+	+
[19]	Ashkenazi Jewish	2	F	Homozygous	c.40_63del	+	+	+	—
[19]	UK	14	UK	Heterozygous	c.40_63del	+	+	—	—
[19]	Spanish	UK	F	Compound heterozygote	c.40_63del	—	+	+	+
[20]	Cypriot	32	F	Heterozygosity	c.2621-2622delGAinsT	+	+	+	+
[20]	Cypriot	UK	M	Heterozygosity	c.3229G>A	+	+	—	—
[6]	Caucasian	8	F	Compound heterozygous	del393G_E131fsX151 2806C>T	+	+	+	—
[6]	Caucasian	35	F	Heterozygous	c443G>T	+	+	—	—
[6]	Caucasian	33	M	Heterozygous	c4981C>T	_	+	—	—
[6]	Caucasian	36	F	Heterozygous	c2083G>A	+	+	+	—
[21]	Chinese	45	M	Heterozygous	c. 2290G >A	+	+	—	—
[22]	Turkish	15	F	Heterozygous	C.2T>C	+	+	+	+
[23]	Italian	13	F	Heterozygous	c.872G>A	+	+	—	—
[24]	African American	14	M	Compound heterozygous	c.4486C>T c.4546C>T	+	+	+	—
[25]	Slovenia	56	F	Heterozygous	c.3547_3548in-sGGA	+	—	—	—
[25]	Slovenia	38	M	Heterozygous	c.1459G>T	+	—	—	—
[26]	Chinese	28	F	Homozygous	c.3725G>A	+	+	+	+
Current study	Chinese	16	M	Heterozygous	c.3227C>T	—	—	+	+
Current study	Chinese	47	F	Heterozygous	c.3227C>T	—	—	+	+
Current study	Chinese	2	M	Heterozygous	c.3227C>T	—	—	—	—

UK: unknown; M/F: male/female; +/-: positive/negative.

## Data Availability

The data used to support the findings of this study are available from the corresponding author upon request.
